# Selection of the Extremes — Male Junior and Adult Ice Hockey Success in relation to Relative Age and its Interaction with Biological Maturation

**DOI:** 10.1186/s40798-025-00902-0

**Published:** 2025-09-08

**Authors:** Erik Niklasson, Marlene Rietz, Oliver Lindholm, John Lind, David M. Johnson, Tommy R. Lundberg

**Affiliations:** 1 Department of Laboratory Medicine, Division of Clinical Physiology, Karolinska Institutet, Stockholm, Sweden; 2 Centre for Physical Activity Research, Rigshospitalet, University of Copenhagen, København, Denmark; 3 Department of Clinical Research, Research Unit OPEN, University of Southern Denmark, Odense, Denmark; 4 Swedish Ice Hockey Association, Stockholm, Sweden; 5 Department for Health, University of Bath, Bath, UK; 6 Football Science and Medicine Department, West Ham United, London, UK; 7 Unit of Clinical Physiology, Karolinska University Hospital, Stockholm, Sweden

## Abstract

**Objectives:**

This study aimed to investigate the relationship between the relative age effect (RAE) and success in ice hockey during adolescence and adulthood in male Swedish players, as well as potential interactions between relative age (RA) and biological maturation.

**Methods:**

Anthropometric data were collected from high schools with a certified ice hockey programme over 20 years. Birth dates were extracted from public databases to calculate numerical relative age (n = 2211 players). Biological maturity timing was defined as the z-score of the percentage of adult height (z%AH) reached at term 1 (~ 16 years of age). Retrospective longitudinal data on selection to junior national teams (U16, U18, U20) and the National Hockey League (NHL) were retrieved from open databases. Junior and adult success probabilities were modelled using generalised logistic modelling (GLM). Spearman correlation analysis was used to assess the correlation between the anthropometric data, relative age, and biological maturation. In addition, the predictor z%AH was added to the GLMs to characterise interactions.

**Results:**

Individuals with a higher relative age were overrepresented in Swedish ice hockey programmes. Players born between January and March (Q1) were about twice as likely to reach the U16 national team as players born between October and December (Q4). Consequently, in a GLM, relative age was identified as a significant predictor of junior success. The addition of z%AH improved model fit for U16 selection, and an interaction between z%AH and RA was observed (*p* < 0.05). In contrast, relative age was not a significant predictor of reaching the NHL (*p* = 0.21). There was no interaction between the RA and z%AH (*p* = 0.44) for adult success. When cross-tabulated, the players most likely to reach both the NHL and the U16 national team were either born early and matured late or born late and matured early.

**Conclusion:**

Early-born and early-maturing players in certified Swedish high school programmes are more likely to be selected for the U16 national team. In terms of adult success, RA had no significant effect on the likelihood of playing in the NHL. However, in a combined model, regardless of relative age, players with late biological maturity timing were more likely to reach the NHL.

**Supplementary Information:**

The online version contains supplementary material available at 10.1186/s40798-025-00902-0.

## Introduction

Youth sports present a complex and dynamic challenge, where various factors contribute to the selection and success of adolescent athletes. Among multiple challenges, two important phenomena associated with grouping athletes by chronological age are inter-individual differences in biological maturation and the relative age effect (RAE).

Differences in biological maturation can lead to a selection bias favouring early maturing young athletes due to their advanced size and physique compared to chronological peers [1, 2]. Indeed, advanced maturation in boys is associated with greater muscle strength, speed, power, and aerobic capacity [3]. This has been shown to impact team-sport specific actions and coach evaluation and ultimately increases the chances of selection [4, 5].

The impact of biological maturation was recently investigated retrospectively in Swedish ice hockey high school programmes. An early maturity bias at the under-16 (U16) national team was reported, but this bias diminished at the U18 and U20 national team levels. Ultimately, a high proportion of the late maturing players reached the National Hockey League (NHL) [6]. This suggests that any initial maturity advantages dissipate over time and do not necessarily translate into long-term success in ice hockey.

On the other hand, the RAE describes an overrepresentation of athletes born early in the chronological selection year due to various advantages associated with relative age [7–10]. While traditionally attributed to physical advantages, recent research shows only minor differences in maturity and fitness between pubertal boys born early and late in the selection year [11]. These findings suggest that the RAE and maturity effects should be considered as largely independent constructs [11–14]. Alternative theories for the RAE propose motivational and developmental processes in which older athletes may benefit from increased perceived competence, self-esteem, coach attention, and other social and psychological opportunities within a selection year [10, 15]. Such benefits, combined with an increased likelihood of an advanced maturity status, could lead to persistent achievement gaps. Additional secondary benefits, such as elite-level selection procedures, could exacerbate these initial differences, leading to a skewed distribution of birth dates among professional athletes [16].

The origins of the RAE in ice hockey date back to 1985 in Canada [7], and have since been well documented. In the NHL, an overrepresentation of players born in the first quarter of the year has been reported at various ages [17–19]. Interestingly, there is some research suggesting that the RAE is lower in the NHL compared to junior leagues [20]. It has even been reported that players born in the last quarter of the year score more points on average and have higher salaries than players born in the first quarter [21]. However, the evidence seems contradictory, with fewer but more impactful late-born players reaching the NHL [20]. Whilst the effects on short- and long-term success by relative age and biological maturity have been studied separately, their potential interaction remains to be explored.

The purpose of this study was to investigate the RAE, both independently and in relation to maturity timing, in Swedish elite ice hockey, examining the distribution of relative age in the junior national teams and players who reached the NHL. We assess the persistence of this effect across different youth selection levels and at the highest elite adult level, using a retrospective longitudinal analysis. In light of our recent publication on the maturity effect within the same cohort [6], we also examined the relationship between the RAE and maturity timing to determine whether they are independent constructs in ice hockey.

## Methods

### Study Population

The Swedish ice hockey structure provides a unique opportunity to analyse biases in talent selection. The country combines certified high school ice hockey programmes with its status as a leading ice hockey nation, creating high-quality longitudinal data through publicly accessible databases that track players from the junior level to the elite adult level. From 1998 until 2017, data was collected from 37 high schools with an ice hockey programme certified by the Swedish Ice Hockey Association. To enter these programmes, players apply for admission during the final year of upper elementary school. The three-year programme begins in the fall when players turn 16 years old and ends in the summer of the year the players turn 19. During this time, the players were subjected to biannual fitness and anthropometrical testing. Data collected from 4787 athletes included in the sample were manually reviewed. To ensure unique identification, players possibly representing multiple clubs were cross-checked using an open-access database [22]. Of the 4787 individuals, 730 and 1846 players had missing data for height at term 1 and term 6 (adult height) of high school, respectively. For 50 players, adult height was recovered from a public database [6]. Female players were excluded due to the low sample size (n = 13).

This retrospective analysis was approved by the Swedish Ethical Review Authority (reference number: 2021–03464). Detailed methods for data extraction, cleaning, and preparation are described in a prior publication [6]. For this article, date of birth (DoB) was extracted for all players with a biological maturation estimate in players in a publicly available database [22].

### Ice Hockey Success

Briefly, players who played for the Swedish junior national teams for males under the age of 16 (Team 16), 18 (Team 18), and 20 years (Team 20) were added using a publicly available database for players born between 1982 and 1998 [23–25]. Selection to National Team 16 was selected as the primary outcome for junior ice hockey success, with selection to Team 18 and Team 20 added as secondary outcomes. Next, information on players in the cohort who played at least one NHL game between the 1998–1999 and the 2023–2024 NHL season was gathered from a publicly available database [26] and from the NHL’s official website [27, 28], updated as of the 11th of February 2024. This variable was used as the primary outcome for adult success.

### Biological Maturation

The estimation of biological maturation has been described previously [6]. Briefly, percentage of adult height (%AH) at term 1 was computed for a sample of 2211 individuals by dividing their height at term 1 by their height at the final term (term 6). For players reaching the NHL with missing height at the final term (N = 40), recovered adult height values were collected from a public database [22]. Among players that reached the NHL and had both term 6 height and recovered adult height, there was no significant difference between the two (N = 56) [6]. Population z-scores of %AH (z%AH) were calculated using a Swedish reference dataset [29] to characterize biological maturation timing. More specifically, z-scores were calculated by subtracting the sex-specific population mean %AH from the %AH of each individual and dividing the result by the corresponding population standard deviation. Additionally, biological to chronological age offset was computed for the elite samples by computing biological age using %AH and reference data [29]. Individuals were grouped into maturity categories (MC) according to quartiles of z%AH, resulting in z-score cut-offs of -0.03, 0.12 and 0.26. The most mature quartile was labelled MC1 (z%AH > 0.26) and the least mature quartile was labelled MC4 (z%AH < -0.03).

### Relative Age Effect

The main focus of this study was on the RAE. Initially, 2109 (95.4%) birthdates were identified for the 2211 players previously included in the biological maturation sample. Players in the sample who did not play in the NHL or any junior national team with missing information on %AH (requiring recorded height at term 1 and adult height) were excluded from the manual DoB extraction. However, the RAE sample was supplemented with data from 254 players who were selected to any junior national team, previously removed due to missing %AH information, as their DoB was available from junior national team records previously compiled. This ensured sufficient statistical power for analyses comparing different elite teams. Therefore, 2363 individuals qualified for inclusion in the RAE sample. A numeric variable describing relative age (RA) across generations of players was computed by using DoB and the cut-off date for selection for the respective age group (31 December) for each player. The difference between the annual cut-off date and DoB was divided by 365.25 (average number of days in a calendar year), resulting in a range of relative age between 0 and 0.99 [30]. A high numerical relative age corresponded to individuals born early in the year. Next, DoB was grouped into categories describing birth months and quarters of birth years, corresponding to January–March (Q1), April–June (Q2), July–September (Q3), and October–December (Q4). Additionally, the number of children born each month in Sweden from January 1st, 1982 until December 31st, 1998 was collected from Statistics Sweden [31].

### Statistical Analysis

As relative age did not follow normal distribution, statistical analysis was conducted using non-parametric testing. The median ± IQR for the sample characteristics was displayed across quarters of birth months, and the proportions of players who reached any elite level were tabulated across quarters of relative age. Next, the association of selection to junior national teams and NHL with numeric RA was assessed using two-sample Wilcoxon or Kruskal Wallis rank sum testing across elite status and between unique elite teams. Next, junior and adult success probabilities were modelled by numeric relative age and relevant confounders using a generalised linear mixed effects (GLME) model with binomial probability distribution function and logit link function. Only individuals with available data for outcome and relevant exposure variables were included. In likelihood ratio testing, the superiority of the GLME over a GLM was tested, initially. For Team 16, the GLME model consisted of Team 16 selection as a binary response variable, and numeric RA and year as fixed effects, in addition to high school region as a random effect. Adjusted intra-class correlation coefficients (ICC) were computed for variance explained by high school region. For the NHL outcome, a singular fit was detected in the GLME, and a simple generalised linear model (GLM) was found superior over a mixed effect modelling approach. The chosen GLM included the binary response variable NHL selection as well as the year of data collection and binary selection to national teams 16, 18, and 20 as fixed effects, as participation in junior national teams likely predisposes individuals to a higher probability of NHL selection. Using likelihood ratio testing, the superiority of the multivariable models over univariable models was confirmed.

To assess the relationship between RA and biological maturation, Spearman’s correlation was performed using RA and %AH, z%AH, and age offset. Maturity categories were tabulated across quarters of RA, and proportions and absolute of players selected to Team 16 or the NHL were visually presented in heatmaps. Lastly, z%AH was added to the prediction models compiled for Team 16 and NHL selection including an interaction with RA, only in individuals with data available for both biological maturation and RA (n = 2109). In sensitivity analyses, height and weight at enrolment to the ice hockey high school (term 1) were included as additional confounders to examine the association of these characteristics on the interaction of RA and biological maturation.

All statistical analysis was performed using R (Version 4.2.3). The packages ggplot2 [32], dplyr [33], ggpubr[34], plotly [35], reshape2 [36], tidyverse [37], lmtest [38], and tidyr [39] were used for data preparation and visualisation, while the glmer or glm function from the lme4 package [40] was employed for modelling.

## Results

### Study Population

For 2363 players, birthdates were registered or could be retrieved retrospectively to estimate RA. Briefly, 602 (25.5%) of the players in the compiled dataset, including relative age, were selected for one of the junior national teams or reached the NHL, of which 368 (15.6%), 351 (14.9%), 330 (14.0%), and 121 (5.1%) players played in national Team 16, Team 18, Team 20, or the NHL, respectively.

Overall, there is a substantially higher relative age in this ice hockey cohort compared to the general Swedish male population born between 1982 and 1998 [31], as shown in Figure S1. Players attending a high school with an ice hockey programme were more likely to be born between January and March (36.7% of the cohort) as compared to October and December (13.2% of the cohort).

In Table 1, sample descriptives are presented across quarters of relative age. Players born early during the year were heavier than players born between October and December (*p* = 0.03) at baseline (term 1), although not significantly taller. Furthermore, there were significant but small differences in %AH between quarters of relative age. Pairwise differences in %AH across most quarters were significant, except for comparisons between Q1 vs. Q2 and Q2 vs. Q3.

**Table 1:** Sample Characteristics at baseline (term 1) and career trajectories across Quarters of Relative Age

		**Q1**	**Q2**	**Q3**	**Q4**	***p***
*RAE*	N (%)	**868 (36.7%)**	**703 (29.8%)**	**481 (20.4%)**	**311 (13.2%)**	*-*
	RA	0.88 ± 0.07	0.63 ± 0.07	0.38 ± 0.07	0.14 ± 0.07	
*Anthropometrics*	Height (m)	1.80 ± 0.06	1.79 ± 0.06	1.79 ± 0.06	1.79 ± 0.06	*0.43*
	Weight (kg)	75.0 ± 7.9	74.1 ± 8.1	74.3 ± 7.7	73.6 ± 8.1	*0.03*
	BMI (kg/m^2^)	23.16 ± 1.88	23.02 ± 1.96	23.07 ± 1.91	22.83 ± 2.01	*0.08*
*Biological Maturation*	%AH (%)	98.9 ± 1.1	98.7 ± 1.3	98.7 ± 1.3	98.3 ± 1.5	< *0.001*
	z%AH	0.10 ± 0.28	0.07 ± 0.32	0.07 ± 0.32	0.01 ± 0.37	< *0.001*
*Career*	Team 16					< *0.001*
	*Yes*	169 (19.5%)	107 (15.2%)	60 (12.5%)	32 (10.3%)	
	*No*	699 (81.5%)	596 (84.8%)	421 (87.5%)	279 (89.7%)	
	Team 18					*0.03*
	*Yes*	152 (17.5%)	90 (12.8%)	61 (12.7%)	48 (15.4%)	
	*No*	716 (82.5%)	613 (87.2%)	420 (87.3%)	263 (84.6%)	
	Team 20					*0.11*
	*Yes*	125 (14.4%)	84 (11.9%)	66 (13.7%)	55 (17.7%)	
	*No*	743 (85.6%)	619 (88.1%)	415 (86.3%)	256 (82.3%)	
	NHL					*0.03*
	*Yes*	44 (5.1%)	25 (3.6%)	27 (5.6%)	25 (8.0%)	
	*No*	824 (94.9%)	678 (96.4%)	454 (94.4%)	286 (92.0%)	

^*^RA is presented as median (IQR) due to non-Gaussian distribution. Other continuous markers are presented as mean (SD). Differences in anthropometrics were analysed using analysis of covariance, and differences in categorical variables were tested using X^2^ statistical testing. *Abbreviations: N – sample size; RAE – relative age effect; RA – relative age; Q – quarter; BMI – body mass index; %AH – percent of adult height at baseline; NHL – National Hockey League*

### Relative Age and Selection

Players who were selected for any elite team (junior national teams and NHL) presented a significantly higher median RA than the non-elite cohort at a median difference of 0.07 (p < 0.001).

**Fig. 1:**
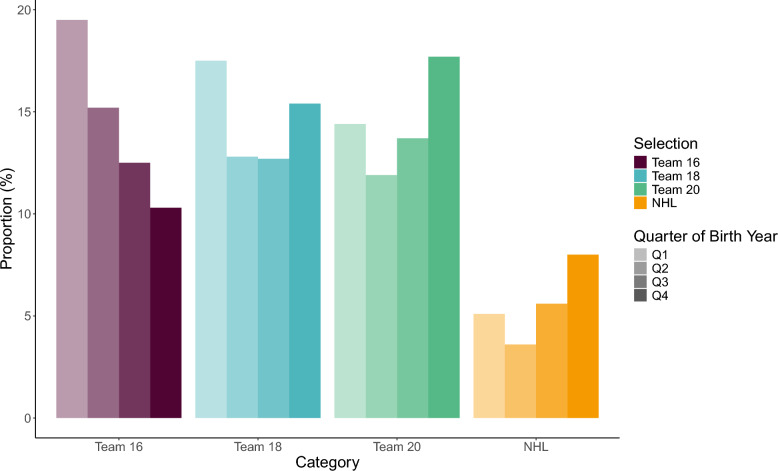
The proportion (%) of players born within each quarter reaching elite levels across different teams. *Abbreviations: Q – quarter; NHL – National Hockey League*

The effect of relative age on selection to elite teams differed across time points of selection. In Fig. 1, the proportion of players selected to unique elite cohorts is presented across quarters of relative age. Significant differences in selection to Team 16, Team 18, and the NHL across birth quarters were found (Table 1). When RA was analysed as a continuous variable, the observed differences in relative age for individuals selected to Team 16 or Team 18 compared to non-selected individuals remained statistically significant, represented as differences in median RA of 0.08 (*p* < 0.001) and 0.06 (*p* < 0.05) for Team 16 and Team 18, respectively.

### Elite Cohorts

**Fig. 2:**
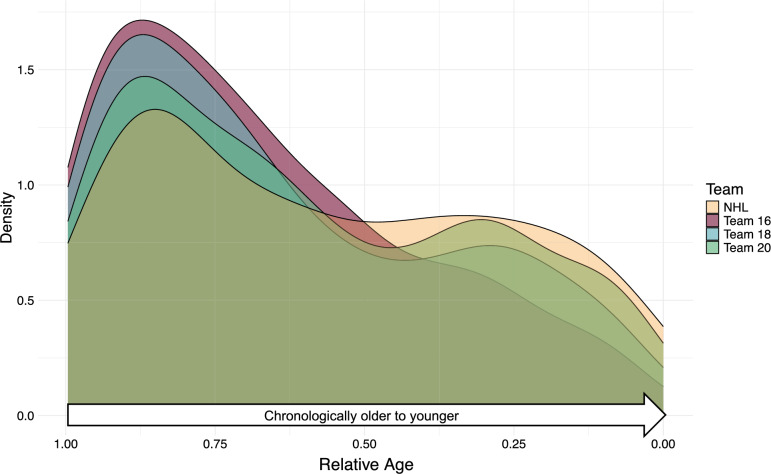
Distribution of Relative Age presenting the absolute number of players across Elite Teams. *Abbreviations: NHL – National Hockey League*

In Fig. 2, density plots showing the distribution of players across RA stratified by elite cohorts are presented. The overrepresentation of early born individuals in the complete sample is represented in the elite sample as a shared mode at an RA of approximately 0.875. While the selection to Team 16 and Team 18 presents a strong preference towards early-born individuals, the trend remains, but flattens, in the older elite cohorts (Team 20 and NHL). Trends regarding biological maturation have been presented previously, but we additionally include a similar density plot for z %AH in Figure S2.

The observed differences in the distribution of RA translated to significant differences in median RA across the four elite cohorts (*p* < 0.05). In pairwise comparisons, significant differences were determined for Team 16 vs. Team 20 and Team 16 vs. NHL comparisons corresponding to 7% and 10% higher relative age in Team 16, respectively (*p* < 0.05).

### Probability of Ice Hockey Success

Overall, 368 (15.6%) individuals with available relative age were selected for the Team 16 national team. To obtain insights into the predictive power of RA concerning this selection, a GLME was performed. This model was superior to a univariate model (*p* < 0.001). In the model, RA displayed a significant association with the selection to Team 16 at an odds ratio (OR) of 2.12 (1.33, 3.39, *p* < 0.01), suggesting approximately doubled chance for Team 16 selection when born on January 01 compared to December 31. Next, 43.0% of the variance in Team 16 selection was explained by the random effect of the high school region (adjusted ICC). Estimated probabilities were graphed across RA for each individual (Figure S3, Table S2).

Moreover, 121 (5.1%) of the players in the sample reached the NHL. The trained GLM outperformed a univariate model (*p* < 0.001), and the removed mixed effect for the region did not significantly contribute to model performance (*p* = 0.991). Briefly, relative age did not significantly impact predicted selection probabilities at an OR (95%CI) of 0.61 (0.28, 1.32, *p* = 0.21, Table S3), although trends favouring players born late in the year were present. Considering other included predictors, the selection to Team 18 (*p* = 0.05) and Team 20 (*p* < 0.001) contributed to the estimated NHL selection probabilities graphed in Figure S4. However, selection to Team 16 did not significantly contribute to the computed chances of reaching the NHL (*p* = 0.47).

### Interaction between Biological Maturation and Relative Age

#### Spearman’s Correlation Testing

Using Spearman’s testing for correlation, significant but trivial correlations between numeric relative age and biological maturation could be determined (Table 2). Scatterplots showing RA across markers of biological maturation are presented in Figures S5–9.

**Table 2:** Spearman’s Correlation between Relative Age and Biological Maturation

	Markers of Biological Maturation	Spearman’s rho	*p*
Relative Age	%AH	0.11	< 0.001
	z-score of %AH	0.07	< 0.01
	Age offset	0.06	< 0.01
	Height	0.03	0.11
	Weight	0.06	< 0.01

*Abbreviations: %AH – percent of adult height at baseline (term 1)*

#### Junior Ice Hockey Success: Team 16

**Fig. 3:**
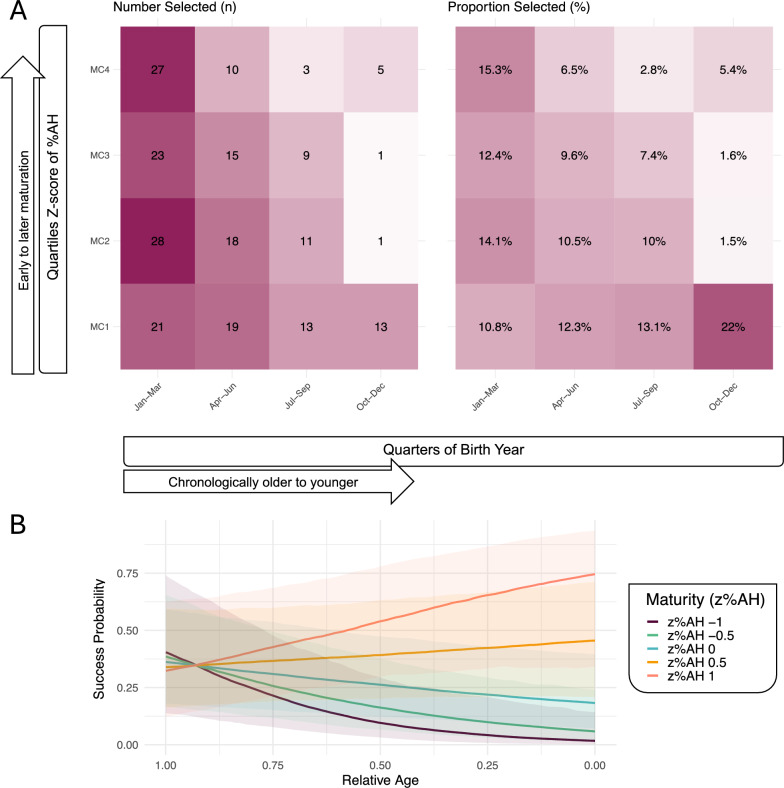
**A.** Number (n) and proportion (%) of Players selected to Team 16 across quarters of RA and maturity categories defined as quartiles of z-scores of %AH, resulting in z-score cut-offs of -0.03, 0.12 and 0.26. **B.** Predicted probability of junior success (national Team 16 selection) as a function of RA and maturity (z%AH) derived from the final GLME model. Predictions were based on a synthetic dataset representing a typical player profile—born in 1996, from the Frolunda region. *Abbreviations: MC – maturity categories defined using quartiles of z%AH, z%AH – Z-score of percent of adult height at baseline (term 1); GLME – generalised linear mixed effects; RA – relative age*

When individuals were grouped across MCs and quarters of their birth year, clear selection patterns could be identified for Team 16 (Fig. 3). Interestingly, the group with the highest proportion of selected players was individuals born late in the year with early biological maturation (22%), although absolute player counts in this group (n = 13) were markedly less than in early born players at any stage of biological maturation, ranging from 21–28 selected players at proportions of 10.8%–15.3%. Furthermore, selection was higher in early matured players and/or players born early in their birth year. Individuals least likely to be selected to the Team 16 were players of moderate biological maturation born at the end of the year.

In the GLME, an interaction term for z%AH significantly improved model fit for the prediction of Team 16 selection (*p* < 0.01), and the interaction was significant (OR [95%CI] 0.06 [0.01, 0.57], *p* < 0.05), see Table S2. In simpler terms, this suggests that the effect of RA on Team 16 selection is dependent on biological maturation and vice versa. For instance, early maturation may make up for low relative age (Fig. 3.A). In the model including biological maturation, the OR (95%CI) for RA changed 2.54 (1.26, 5.12, *p* < 0.01), and the OR for biological maturity timing suggested stronger effects of biological maturation than relative age (13.29, 95%CI: 2.69, 65.58, *p* < 0.01). In Fig. 3.B, the predicted probability for Team 16 selection computed with the chosen model was graphed as a function of RA and z%AH. Lastly, 46.5% of variance in the outcome were explained by the random effect of high school region (Table S2).

When height and weight at baseline (term 1 of the ice hockey high school) were added during sensitivity analysis, the interaction between RA and biological maturation (*p* < 0.05), as well as the OR of RA (*p* < 0.01) and biological maturity timing (*p* < 0.01) remained significant, with an added association of weight [OR estimate 1.05 (95% CI: 1.03, 1.08, *p* < 0.001)], but not height *(p* = 0.36). See Table S2 for fixed effect coefficients, ORs, and the adjusted ICC.

#### Adult Ice Hockey Success: NHL

**Fig. 4:**
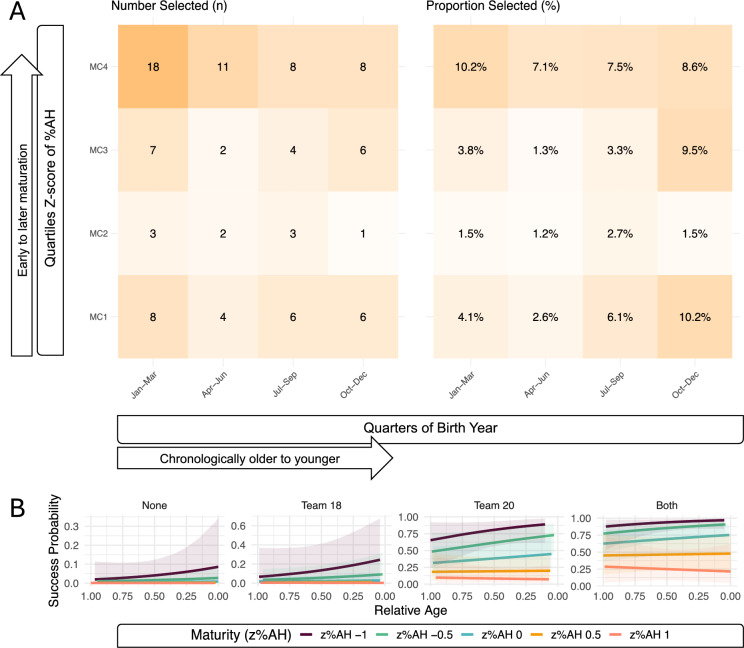
**A.** Number (n) and proportion (%) of players reaching the NHL across quarters of RA and maturity categories defined as quartiles of z-scores of %AH, resulting in z-score cut-offs of -0.03, 0.12 and 0.26. **B.** Predicted probability of adult success in the NHL as a function of RA and maturity (z%AH), stratified by elite youth team participation, derived from the final GLM, see 2.5 Statistical analysis. Predictions are based on a synthetic dataset representing a typical player profile—born in 1996, from the Frolunda region, and without experience in the National Team 16. The figure displays model-predicted probabilities with 95% confidence intervals (shaded ribbons) across four categories of elite youth involvement: no participation in Teams 18 or 20 (“None”), participation in Team 18 only, Team 20 only, or both teams (“Both”). *Abbreviations: MC – maturity categories defined using quartiles of z%AH; z%AH – Z-score of percent of adult height at baseline (term 1); GLM – generalised linear model; RA – relative age*

When assessing adult ice hockey success, NHL selection patterns (Fig. 4.A) differed from the observed trends in junior ice hockey success. Players with the latest biological maturation timing (MC4) presented the highest proportion of players reaching the NHL, no matter when they were born. Next, the proportion of selected players born in MC1 and belonging to the earliest maturity quartile was equal to the proportion of players with late maturation born in Q1 (10.2%), although the absolute number in the former category was three times as high. The combination of moderate maturation timing with moderate relative age seemed to be the least beneficial combination of predictors for NHL success.

Next, these trends were assessed by adding an interaction for z-scores of %AH and RA to the GLM for the estimation of NHL selection probabilities. Adding biological maturation as a predictor significantly improved model fit (*p* < 0.001), but the interaction between RA and z%AH was insignificant (OR 2.69, 95%CI: 0.21, 34.71, *p* = 0.44), see Table S3. Similar to the simple model, the model including biological maturation did not suggest a significant impact of RA on NHL selection at an OR estimate (95%CI) of 0.55 (0.2, 1.48, *p* = 0.24). However, a significant association was determined for z%AH [OR estimate (95%CI) of 0.09 (0.02, 0.38, *p* < 0.01)], and the selection to Team 18 (*p* < 0.01) and Team 20 (*p* < 0.001) remained significant predictors within the model. In simpler terms, this means that the observed trends of late biological maturation favouring NHL selection seem to be independent of RA in the given model. In Fig. 4.B, the predicted probability for NHL selection computed with the chosen model was graphed as a function of RA and z%AH.

When height and weight in the first term of ice hockey school were added during sensitivity analyses, the interaction between relative age and biological maturation timing remained insignificant *(p* = 0.62). In this model, the OR (95%) for RA was 0.42 (0.15, 1.18, *p* = 0.10), suggesting present though insignificant trends of lower RA favouring NHL selection. The OR of biological maturation remained significant at an OR of 0.05 (95%CI: 0.01, 0.24*, p* < 0.001) in addition to a significant OR (95%CI) for height (10 cm) at an estimate of 2.83 (1.37, 5.85, *p* < 0.01), and no impact of weight *(p* = 0.94). This emphasises that the inclusion of these variables in the model decouples the associations of biological maturation and relative age from trends simply attributable to height and weight, thereby strengthening their associations with NHL selection. Comparing two individuals with the same biological maturity, relative age, and birth year, the OR for height suggest that a 10 cm difference in height is associated with nearly tripled odds of NHL selection in the given model.

## Discussion

This study provided insight into the extent to which relative age, independently and in combination with maturity timing, is associated with junior and adult ice hockey success in a Swedish retrospective cohort. We report a strong RAE for selection to the national Team 16, while the RAE was not associated with playing in the NHL. An interaction between relative age and maturity timing was suggested in national Team 16, while maturity-related associations with NHL probabilities were independent of RA. Striking tendencies to select players at the extremes of relative age and biological maturity were observed both during selection for Team 16 and in predicting who will play in the NHL (adult success).

Previously, we reported a selection bias in national Team 16 favouring early maturing players for selection, and in contrast, we showed that late maturing players were more likely to reach the NHL in this sample [6]. The relative age data in the current study largely reflect these trends: A strong RAE was present at the Team 16 level, and decreased towards Team 18 selection, although it was still significant. On the other hand, adult success, i.e. reaching the NHL, was largely independent of the RAE. Nonetheless, whilst the absolute number of NHL-selected players born in Q4 was similar to Q2 and Q3, Q4 had the highest proportion of players reaching the NHL. While the observed results are consistent with previous studies reporting a reversal, or at least attenuation, of the RAE in professional ice hockey with increasing age [20, 21, 41], our data suggest that there is a persistent advantage of the RAE, with an increased number, but not proportion, of players born early reaching the NHL. Although the effect diminishes over time as the distribution becomes flatter, it is not fully neutralized from the junior national teams to the NHL.

The findings also align with the "underdog hypothesis", which states that a player who is skilled enough to overcome early selection biases, here represented by the recruitment to certified ice hockey programmes, is likely to remain successful over time [41]. The highly skewed distribution of birth dates at baseline suggests that many late-born players may not get the same development opportunities if removed from the talent pool before the age of 16. Consequently, a significant fraction of future talent may be lost in this selection step. Compared to previous studies reporting an RAE in the U20 ice hockey World Championships [42], our data suggest that the RAE present in the Swedish teams is largely a reflection of the available players to select from in certified ice hockey programmes. However, an RAE has also been reported in Swedish children younger than six years, including cross-country skiing and orienteering sports [43]. In Swedish female ice hockey, an RAE was observed at the age of five [44], and in unpublished data from the Swedish ice hockey player registration system, skewed relative age towards early born individuals was observed at the age of eight. This suggests that differences in relative age may also be due to factors influencing early dropout and sport selection.

Interestingly, we observed an interaction between relative age and maturation in the Team 16 selection. The results suggest that the previously reported bias toward early-maturing players may, in part, be influenced by relative age [6]. In short, early-born, early-maturing players represented the largest absolute number of players selected to Team 16. This reflects the common advantage of being both physically advanced and older within the cohort [8]. However, there is a notable proportion of players selected for Team 16 who were born late but matured early. This suggests that advanced biological maturity may compensate for the disadvantage of a later birth date, as previously reported [6]. In contrast, players in certified ice hockey high school programmes who were late in their maturity timing were more likely to reach the NHL, regardless of relative age. Here, this trend shifts in favour of later maturing players, underscoring that long-term potential may not be fully realized through early selection processes [45].

An interesting observation was the selection pattern of favouring players at the extremes of both relative age and biological maturation. This was true for both Team 16 and the NHL, as the players most likely to participate at both levels were those born in Q1 with late maturation [*Proportion selected*, Team 16: 15.3% | NHL: 10.2%] and players born in Q4 with early maturation [*Proportion selected*, Team 16: 22%| NHL: 10.2%]. However, the strength of the association between ice hockey success and biological maturity timing was consistently greater than that of RA. Indeed, the observed biases give rise to concerns about how well youth ice hockey handles the relative age and maturity effects. Ideally, neither a player's birth month nor their timing of maturation should influence future potential and the likelihood of succeeding at the elite level as an adult. Overall, regular monitoring of growth and maturation should be an integral part of elite ice hockey development programs. Ice hockey organizations should also recognize the limitations of their current selection processes and work towards a more inclusive system that maximizes the long-term potential of all players rather than reinforcing short-term gains. As shown in this study, a key challenge is to differentiate between early maturity timing and expected adult height, as these factors have opposite effects on a player’s likelihood of adult success. Therefore, scouts/coaches should consider biological maturation and relative age when selecting players in the elite environment to better understand each player’s potential in relation to their current performance level.

Promising but largely unproven interventions for ice hockey organisations may include birthday banding [46] and bio-banding [8, 47] to account for relative age and maturity timing, respectively. Shirt numbering by relative age or maturity is an additional strategy to visually remind coaches and scouts of unconscious selection bias [48, 49]. In bio-banding, athletes are grouped by biological maturity rather than chronological age [3, 8]. This strategy was recently tested in ice hockey. It was reported that both late and early maturing players benefitted from the intervention [50]. Lastly, parallel selection processes, such as the "future team" concept, accommodating late-maturing players, may be particularly useful at the Team 16 level [51, 52]. As our results indicate, selection to the national Team 16 does not accurately predict long-term success. Therefore, creating alternative development pathways for late-born or late-maturing players may prevent the premature narrowing of talent development pipelines.

This study has several strengths. To our knowledge, this cohort is the largest resource combining biological maturation and RA in a sports context, allowing for a longitudinal analysis. The included national references for birth dates [31] and height [29] further supported this aim. Similarly, by following players longitudinally and including non-elite players, we were also able to examine how RA and biological maturity timing influence selection over time. However, all included players had been selected into high school ice hockey programmes. These programmes involve a rigorous selection process, resulting in a sample skewed toward players with early birthdates and early maturation compared to the general Swedish population, which may limit generalisability. When interpreting the study, readers should also be aware of the use of both absolute and relative success rates. Finally, the results may not be directly transferable to other sports or to female athletes and future research should examine how these selection dynamics operate in other contexts. Future studies should also investigate how factors such as biological sex, physical demands of the sport, and degree of early specialisation influence selection outcomes. In addition, the field would benefit from more frequent and extended longitudinal monitoring of players' anthropometric data to better capture the effect of RA and biological maturation and their interaction on selection biases.

## Conclusion

In conclusion, we report a pronounced selection bias in youth ice hockey favouring early-born players when it comes to selection for the junior national team 16, especially if they mature early. However, later maturing players were more likely to make the NHL (adult success), regardless of their relative age. Collectively, there were clear tendencies towards selecting extremes in relative age and biological maturation, which underscores the importance of understanding these dynamics to better develop junior ice hockey players.

## Ethics approval

The Swedish Ethical Review Authority, Access Number: 2021–03464.

## Competing Interests

TRL and OL have received financial compensation from the Swedish Ice Hockey Association for consultancy work. EN and MR have received reimbursement of travel expenses from the Swedish Ice Hockey Association. JL is employed by the Swedish Ice Hockey Association. DJ declares no competing interests.

## Supplementary Information

Additional file1 (PDF 1269 KB)

## Data Availability

Selected data are available upon reasonable request from the corresponding author. The data are not publicly available because combinations of variables could compromise the identities of individual participants.
