# Supplementary material for: Selection of the Extremes — Male Junior and Adult Ice Hockey Success in relation to Relative Age and its Interaction with Biological Maturation
Source: Sports Med Open. 2025 Sep 8;11:102. doi: 10.1186/s40798-025-00902-0 (PMC12417341; doi:10.1186/s40798-025-00902-0)
Supplement: Supplementary file 1 — Additional file1 (PDF 1269 KB) [file 40798_2025_902_MOESM1_ESM.pdf]

Supplementary Material

Selection of the Extremes — Male Junior and Adult Ice Hockey Success in Relation to Relative Age and its Interaction with Biological Maturation

Erik Niklasson[1,2*], Marlene Rietz[1,3*], Oliver Lindholm[1] , John Lind[4], David M. Johnson[5, 6], Tommy R Lundberg, PhD[1,7]

AFFILIATIONS
[1] Department of Laboratory Medicine, Division of Clinical Physiology, Karolinska Institutet, Stockholm, Sweden
[2] Centre for Physical Activity Research, Rigshospitalet, University of Copenhagen, Denmark
[3] Research Unit OPEN, Department of Clinical Research, University of Southern Denmark, Odense, Denmark
[4] Swedish Ice Hockey Association, Stockholm, Sweden
[5] Department for Health, University of Bath, Bath, UK
[6] Football Science and Medicine Department, West Ham United, London, UK
[7] Unit of Clinical Physiology, Karolinska University Hospital, Stockholm, Sweden

*Authors contributed equally and share first authorship.

Corresponding author:
Tommy R. Lundberg, PhD
Email: tommy.lundberg@ki.se
Department of Laboratory Medicine
Division of Clinical Physiology, ANA Futura
Karolinska Institutet
14152 Huddinge, Sweden

Supplementary Table 1

Median (IQR) Relative Age across Elite Teams

Teams	Team 16	Team 18	Team 20	NHL
Median (IQR)	0.72 (0.37)	0.70 (0.46)	0.65 (0.52)	0.62 (0.53)

Group-wise Comparisons: Kruskal-Wallis Rank Sum

Comparison	Z statistic	p (Bonferroni)
Team 16 – Team 18	1.30	0.58
Team 16 – Team 20	3.10	<0.01*
Team 18 – Team 20	1.80	0.21
NHL – Team 16	-3.16	<0.01*
NHL – Team 18	-2.22	0.08
NHL – Team 20	-0.90	1.00

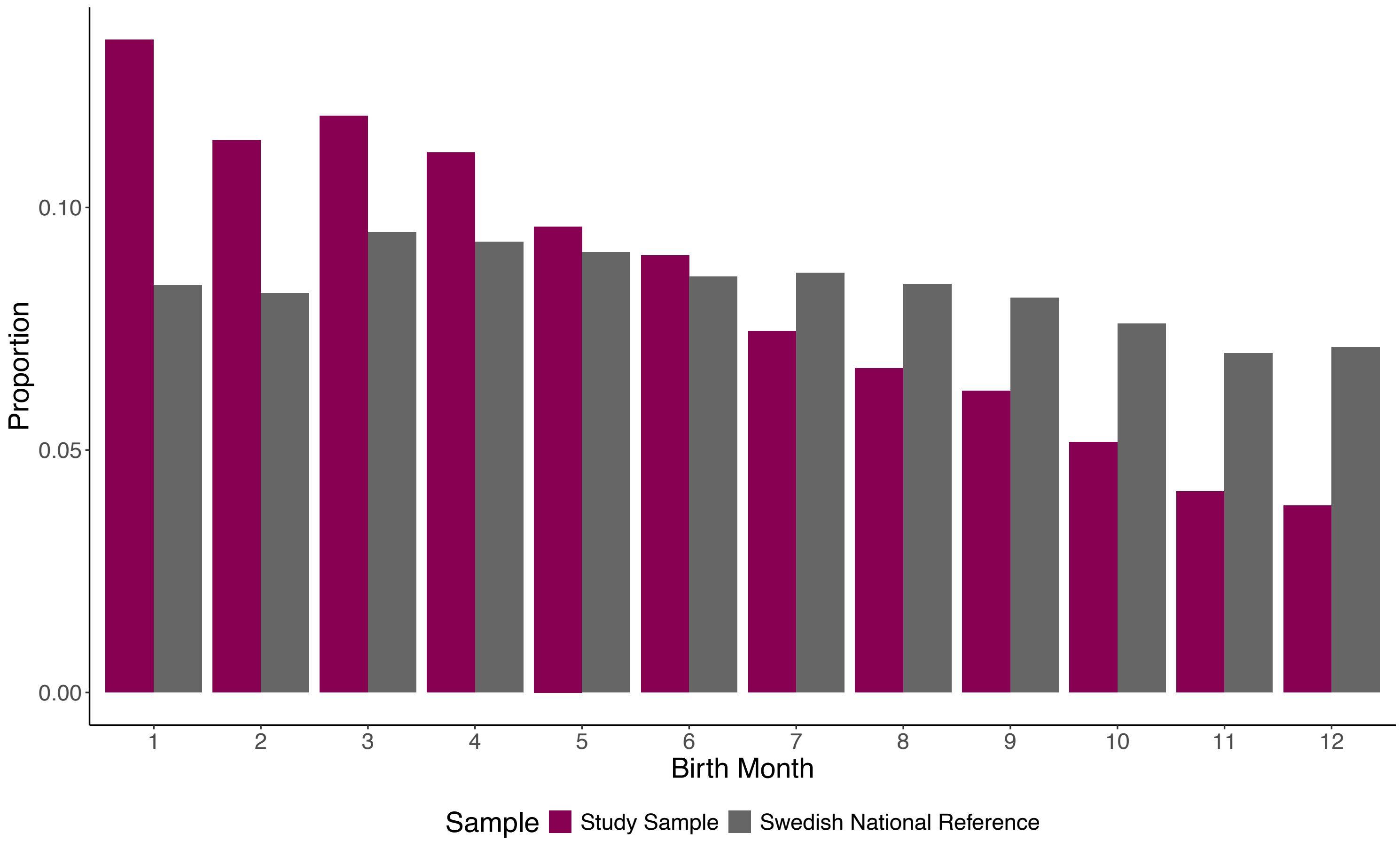

Supplementary Figure 1. Proportions of individuals born in each birth month comparing this study sample with a Swedish National Reference for the birth years 1982–1998.

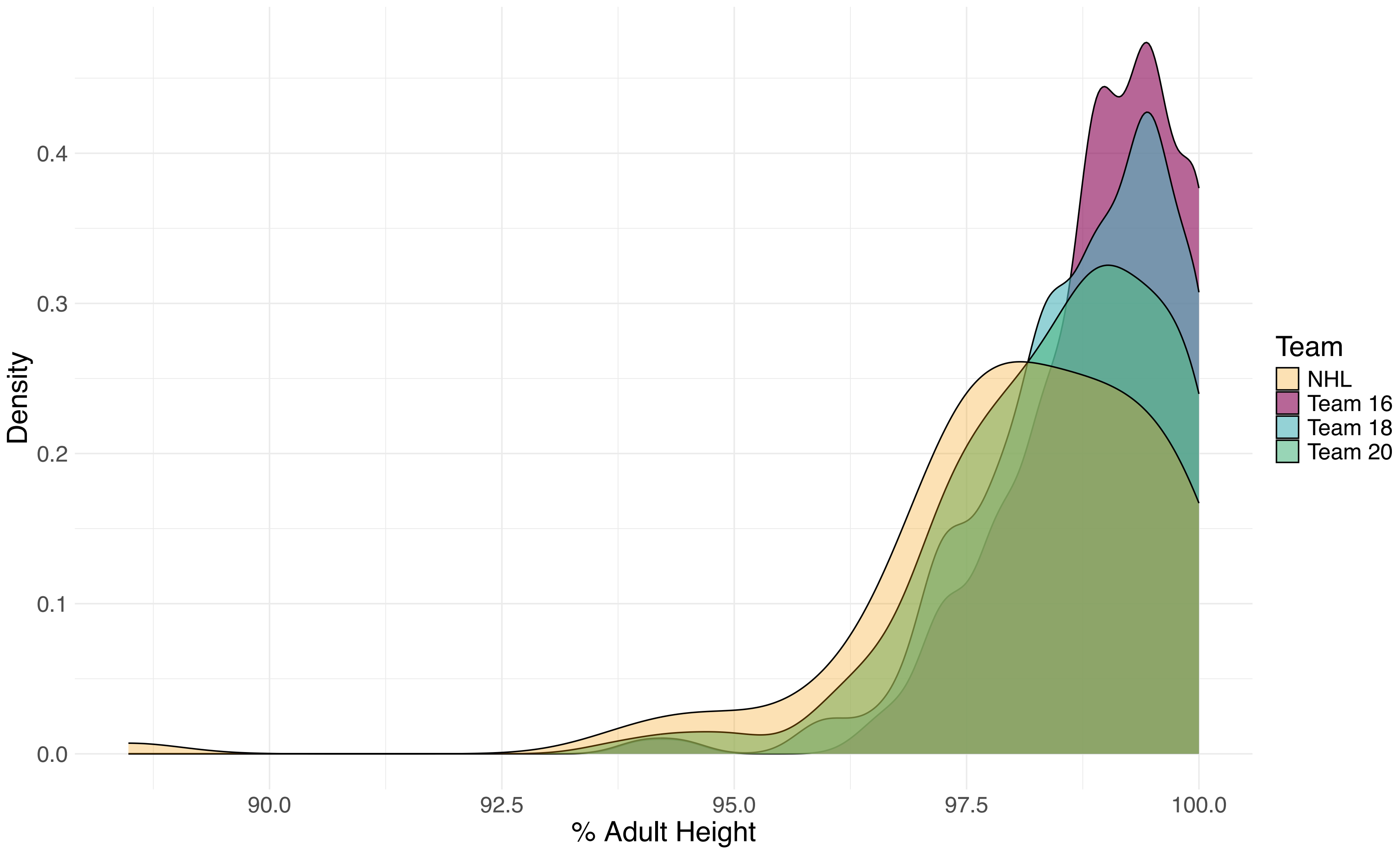

Supplementary Figure 2. Distribution of %AH across Elite Teams.

Supplementary Table 2. Coefficients and Odds Ratios for Team 16 Selection

Team 16		Coefficient (95% CI)	Odds Ratio (95% CI)	p	Adjusted ICC
Model Simple	RAE	0.75 (0.28, 1.22)	2.12 (1.33, 3.39)	0.00168	0.430
Model Interaction	RAE	0.93 (0.23, 1.63)	2.54 (1.26, 5.12)	0.0091	0.465
	z%AH	2.59 (0.99, 4.18)	13.29 (2.69, 65.58)	0.00149	
	RAE:z%AH	-2.78 (-5.00, -0.56)	0.06 (0.01, 0.57)	0.01405	
Sensitivity	RAE	0.94 (0.23, 1.65)	2.56 (1.26, 5.21)	0.0094	0.430
	z%AH	2.23 (0.59, 3.86)	9.27 (1.81, 47.5)	0.00755	
	RAE:z%AH	-2.73 (-4.99, -0.46)	0.07 (0.01, 0.63)	0.01828	
	Weight (kg)	0.05 (0.03, 0.08)	1.05 (1.03, 1.08)	0.00016	
	Height (10 cm)	-0.17 (-0.52, 0.19)	0.84 (0.63, 1.20)	0.36012	

For Team 16, the GLME model consisted of Team 16 selection as a binary response variable, and numeric RAE and birth year as fixed effects, in addition to high school region as a random effect. Here, coefficients and odds ratios including 95%CIs are presented for a simple model, a model including the interaction of RAE and z%AH, as well as sensitivity analysis using the interaction model, but including weight (kg) and height (10 cm) at term 1 of the ice hockey high school programme. *Abbreviations: CI – confidence interval; RAE – relative age effect; z%AH – Z-score of percent of adult height at baseline (term 1); ICC – intra-class correlation coefficient for the random effect of high school region.*

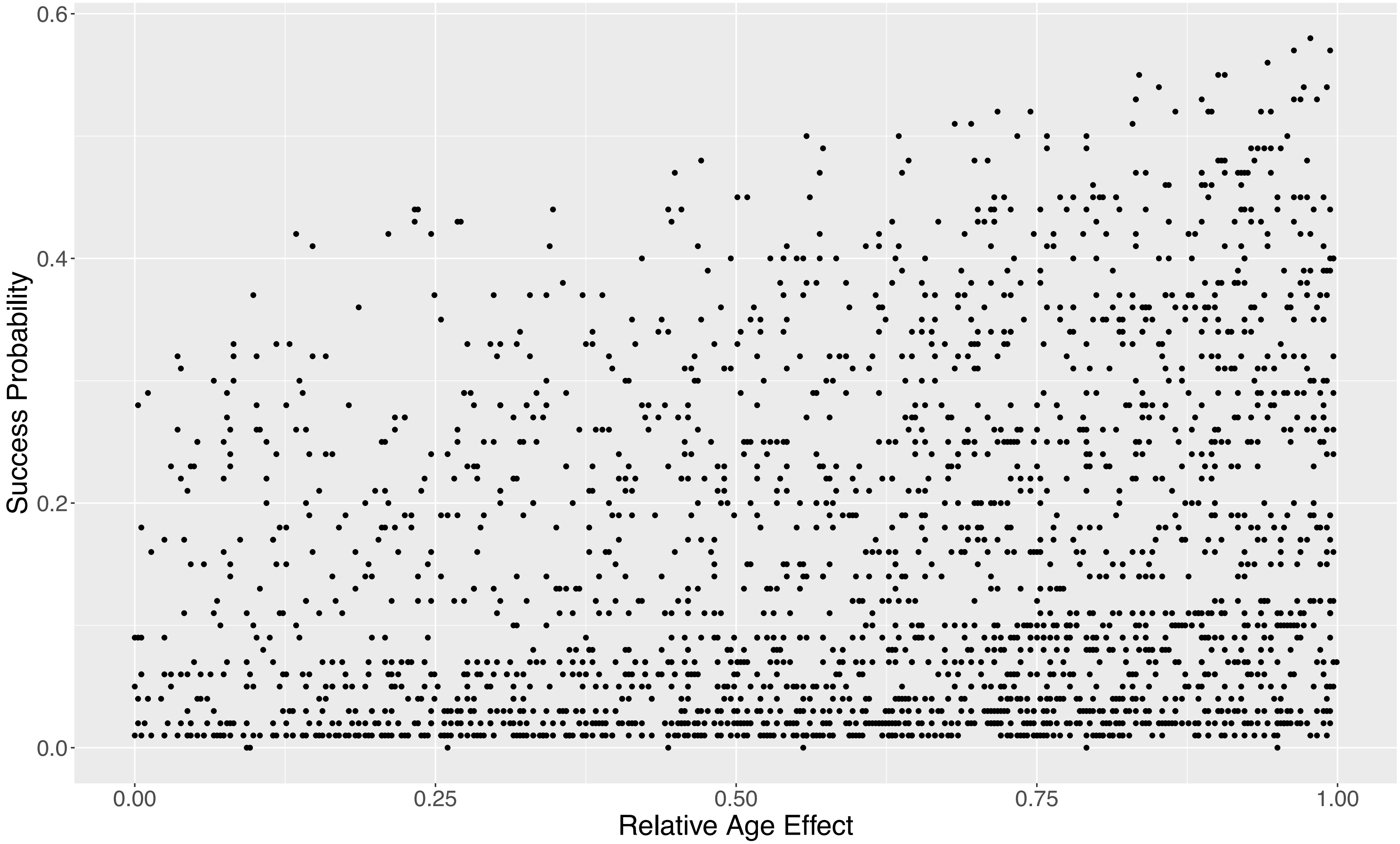

Supplementary Figure 4. Estimated Team 16 Success Probabilities across Relative Age Effect.

Supplementary Table 3. Coefficients and Odds Ratios for NHL Selection

NHL		Coefficient (95% CI)	Odds Ratio (95% CI)	p
Model Simple	RAE	-0.5 (-1.28, 0.27)	0.61 (0.28, 1.32)	0.20568
Model Interaction	RAE	-0.6 (-1.6, 0.39)	0.55 (0.2, 1.48)	0.23567
	z%AH	-2.41 (-3.84, -0.97)	0.09 (0.02, 0.38)	0.00103
	RAE:z%AH	0.99 (-1.57, 3.55)	2.69 (0.21, 34.71)	0.44879
Sensitivity	RAE	-0.87 (-1.9, 0.16)	0.42 (0.15, 1.18)	0.09862
	z%AH	-2.96 (-4.48, -1.43)	0.05 (0.01, 0.24)	0.00015
	RAE:z%AH	0.69 (-2.01, 3.38)	1.99 (0.13, 29.37)	0.61755
	Weight (kg)	0.00 (-0.06, 0.06)	1.00 (0.95, 1.06)	0.93653
	Height (10 cm)	1.04 (0.32, 1.77)	2.83 (1.37, 5.85)	0.00489

For the NHL, a simple generalized linear model (GLM) was found superior over a mixed effect modelling approach. The chosen GLM included the binary response variable NHL selection as well as the birth year and binary selection to national teams 16, 18, and 20 as fixed effects, as participation in junior national teams likely predisposes individuals to a higher probability of NHL selection. Here, coefficients and odds ratios including 95%CIs are presented for a simple model, a model including the interaction of RAE and z%AH, as well as sensitivity analysis using the interaction model, but including weight (kg) and height (10 cm) at term 1 of the ice hockey high school programme. *Abbreviations: CI – confidence interval; RAE – relative age effect; NHL – national hockey league; z%AH – Z-score of percent of adult height at baseline (term 1)*

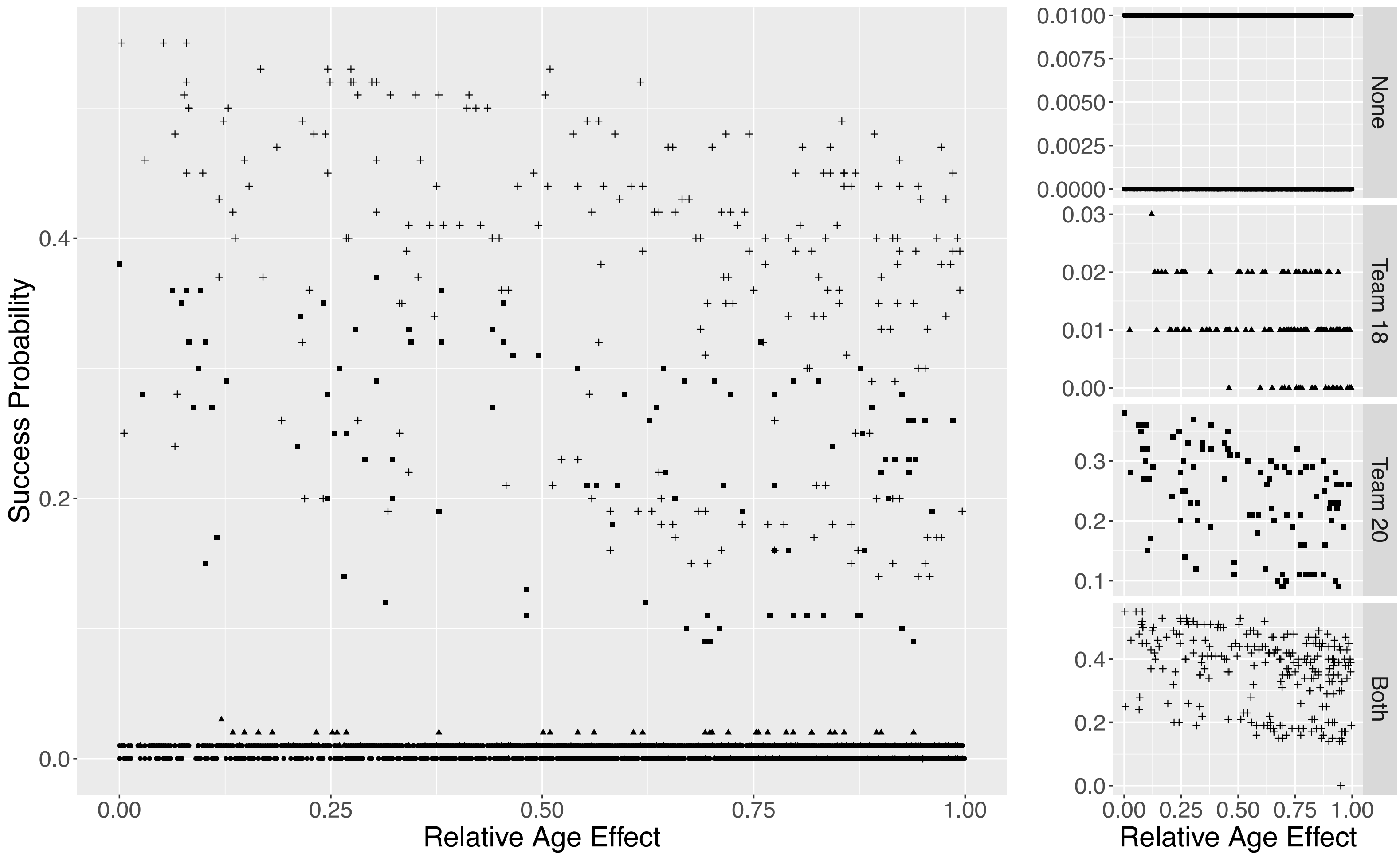

Supplementary Figure 4. Estimated adult Success (NHL) Probabilities across Relative Age Effect.

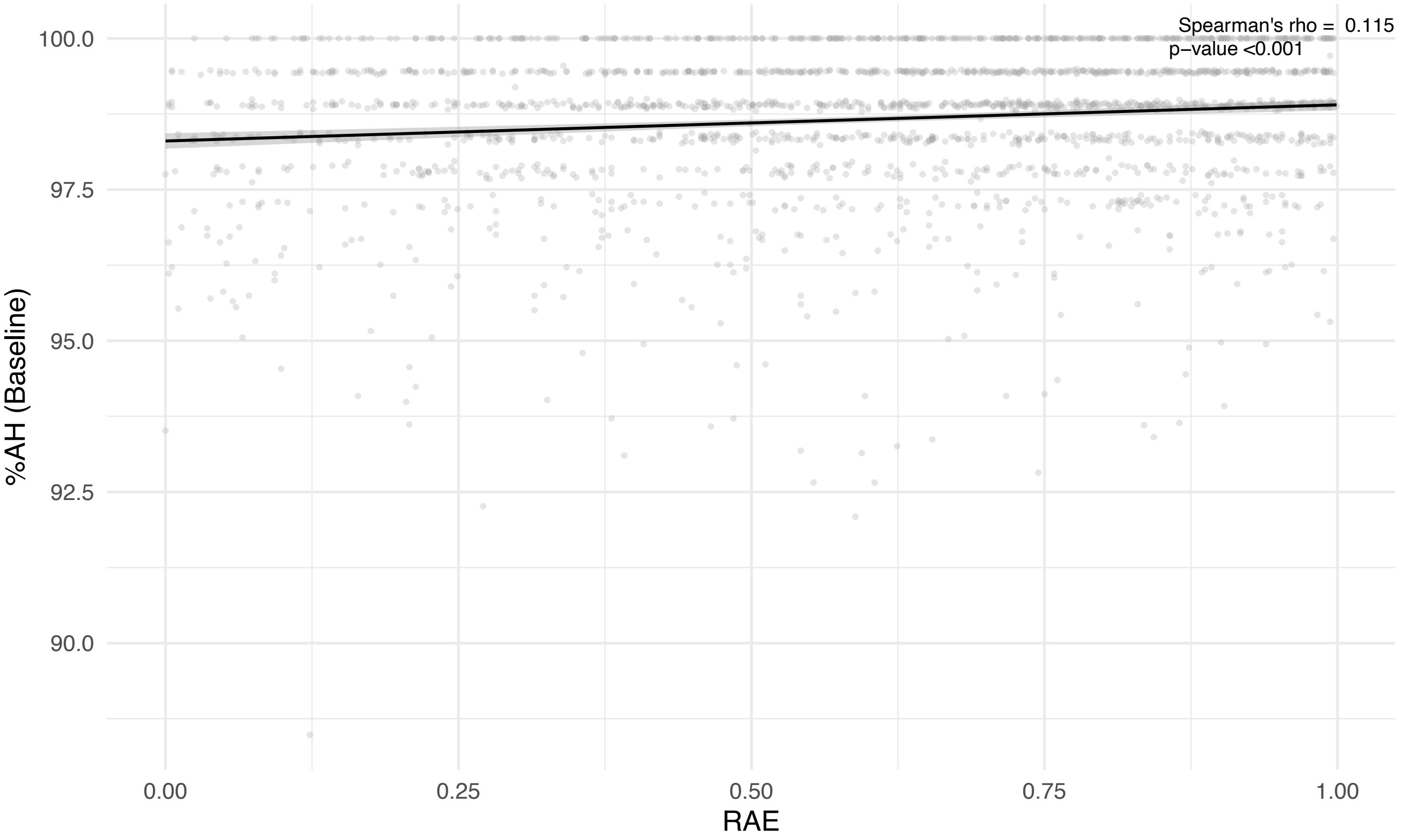

Supplementary Figure 5. Scatterplot of RAE across %AH. | *Abbreviations: RAE – relative age effect*

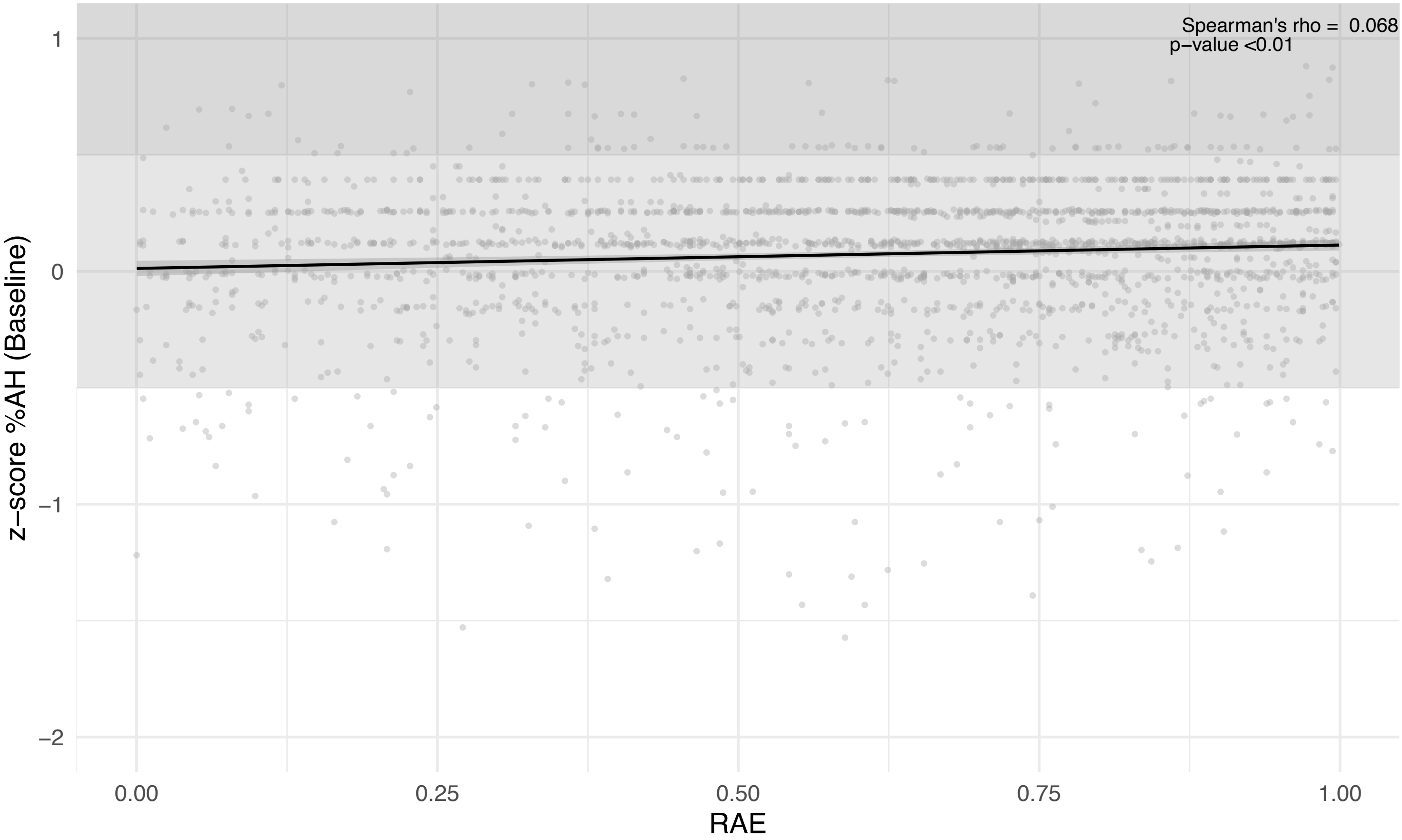

Supplementary Figure 6. Scatterplot of RAE across z-scores of %AH. | *Abbreviations: RAE – relative age effect*

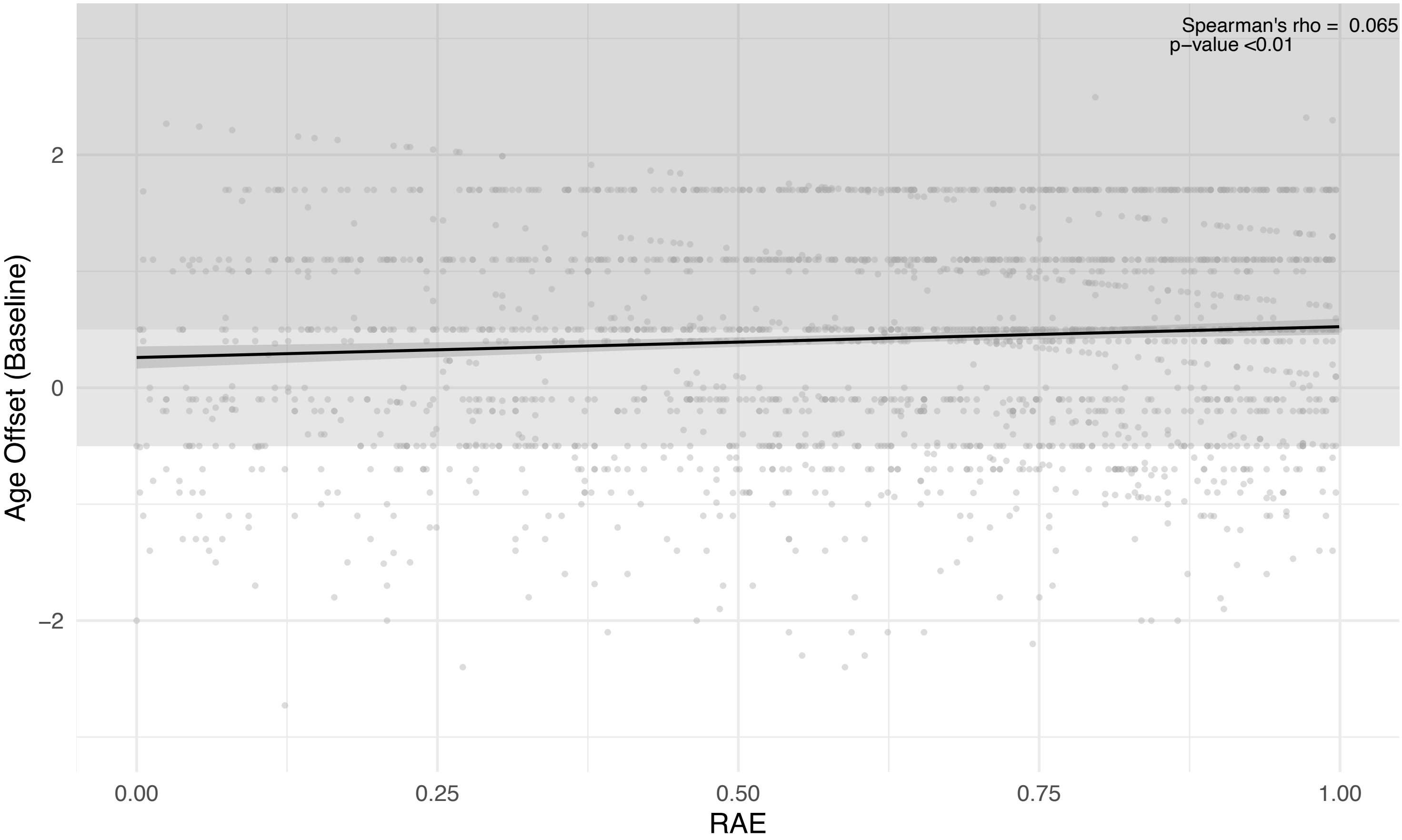

Supplementary Figure 7. Scatterplot of RAE across age offset. | *Abbreviations: RAE – relative age effect*

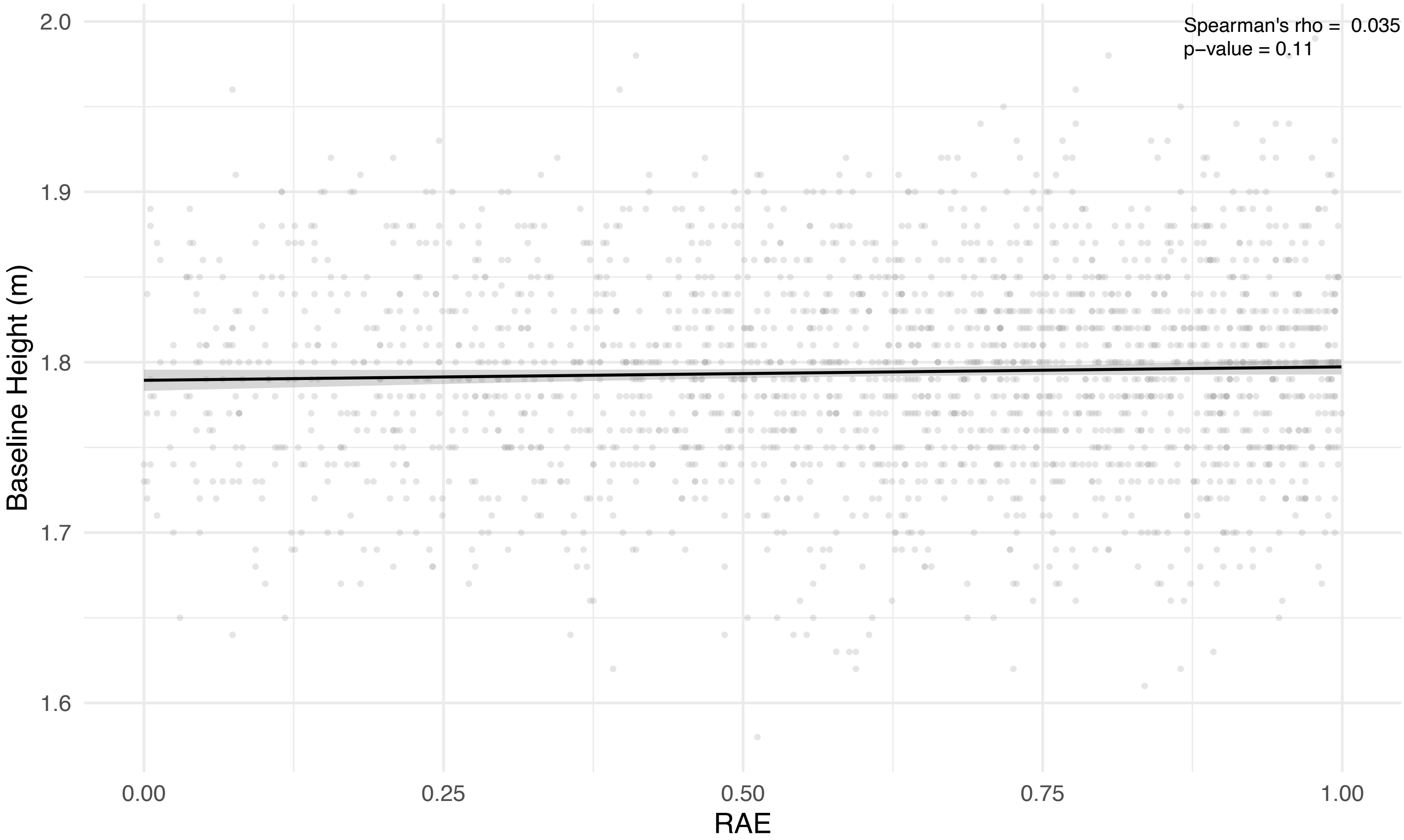

Supplementary Figure 8. Scatterplot of Height (m) across age offset. | *Abbreviations: RAE – relative age effect*

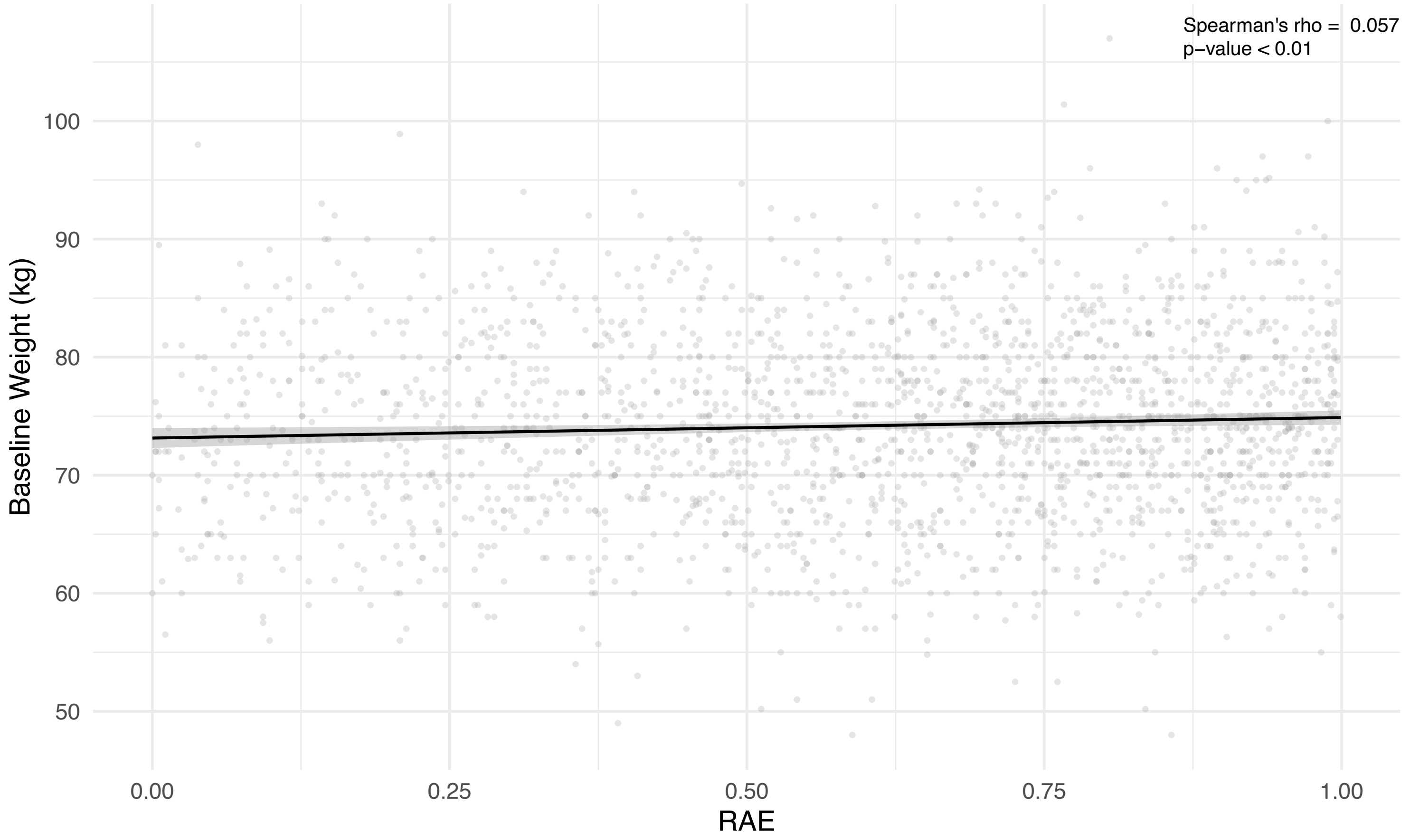

Supplementary Figure 9. Scatterplot of Weight (kg) across age offset. | *Abbreviations: RAE – relative age effect*
